# Long-Term Sequelae of Primary Tooth Injuries on Permanent Successors: A Case Series

**DOI:** 10.7759/cureus.107991

**Published:** 2026-04-29

**Authors:** Mridula Goswami, Akansha Gupta, Gyanendra Kumar, Riya Marie Johnson, Farheen Sultan, Rimshheanam Rimshheanam

**Affiliations:** 1 Pediatric and Preventive Dentistry, Maulana Azad Institute of Dental Sciences, New Delhi, IND

**Keywords:** deciduous tooth, enamel hypoplasia, pediatric, permanent tooth, sequelae, tooth eruption, tooth injuries

## Abstract

Trauma to the primary dentition is a common occurrence in early childhood and poses a significant risk to the developing permanent successors due to their close anatomical relationship. The sequelae may have delayed presentation, where symptoms may arise even years after the trauma in the form of developmental disturbances to the permanent teeth. This case series presents five children who presented with developmental sequelae to their permanent successors after traumatic injury to their predecessor primary teeth. The presentations in the cases included enamel hypoplasia, crown dilaceration, delayed eruption, impaction, odontome-like formations, and pulpal pathologies, which required multidisciplinary management including endodontic, orthodontic, surgical, and restorative intervention based on the individual presentation of the developmental disturbance. Primary tooth trauma can have diverse sequelae in the permanent successors with delayed presentation, and requires multidisciplinary management, early diagnosis, and long-term follow-up to ensure the best functional and esthetic outcomes.

## Introduction

Traumatic dental injuries (TDIs) are highly prevalent in childhood, with nearly half of all children experiencing at least one injury by 18 years of age [1-3]. TDIs in the primary dentition occur most frequently during early childhood due to immature motor coordination and an increased risk of falls, as shown by global and Indian prevalence rates of 22.7% and 24.2%, respectively [4,5]. Falls (43%) constitute the predominant etiological factor for TDIs in the primary dentition in India, followed by sports-related injuries (26%) and collisions (12%). Road traffic accidents account for 8% of cases, whereas violence and other causes each contribute approximately 7% [6,7]. The maxillary central incisors are most commonly affected, accounting for 73.9% of cases owing to their prominent position in the arch, and luxation injuries predominate (41.8%), followed by avulsion (19.4%), with root and crown fractures each representing 5.8%, concussion 3.7%, and isolated fractures 2.9% [7,8].

Sequelae in permanent teeth following trauma to the primary dentition were reported in 12-74% of cases and largely influenced by the close anatomical proximity of primary root apices to the developing permanent tooth germs, which may be broadly classified into two categories, early and long-term [9]. Early sequelae are detectable radiographically within six to 12 months, while long-term sequelae manifest clinically during eruption of the permanent successors, usually two to six years post-trauma [10].

Management of post-traumatic sequelae can be time-consuming and require a multidisciplinary approach. Due to the wide spectrum of clinical presentations and developmental disturbances following trauma to the primary dentition, no single treatment protocol is universally applicable; therefore, this case series highlights the need for individualized, case-specific management to achieve optimal functional and esthetic outcomes.

This report aims to describe five representative cases of long-term sequelae in permanent successors following primary tooth trauma, outline the diagnostic pathway used, and discuss multidisciplinary management strategies and follow-up intervals. Cases were selected based on the presence of clinically and radiographically evident developmental sequelae in permanent successors with a documented history of trauma to the corresponding primary predecessors. Patients with developmental anomalies unrelated to trauma or inadequate follow-up records were excluded.

## Case presentation

The present case series shows five pediatric cases presented with developmental sequelae in the permanent teeth secondary to trauma sustained by their primary predecessors during early childhood (Table 1).

**Table 1:** Demographic details, diagnosis, and treatment plan of all cases F: Female; M: Male; MBT: McLaughlin, Bennett, and Trevisi; MTA: Mineral Trioxide Aggregate; GIC: Glass Ionomer Cement. Manufacturer details: Glass Ionomer Cement (3M, St. Paul, MN, USA); Bioceramic Sealer (Meta Adseal Plus, META BIOMED Co., Ltd., Cheongju-si, Korea); Diode Laser (Epic X Diode Laser, BIOLASE, Irvine, CA, USA).

Case	Age/Sex	Trauma history	Time since trauma	Type of primary tooth injury	Sequelae in permanent tooth	Diagnosis	Management
1	11/F	Fall at age of 3 years; avulsion of 51 & 61	8 years	Avulsion	Crown dilaceration + hypoplasia of 21	Chronic irreversible pulpitis in 21	Clinical and radiographic examination followed by access opening in 21. Working length determined radiographically. Cleaning and shaping performed with intracanal Calcium hydroxide dressing placed as interim medicament. Due to altered canal anatomy, modified gutta-percha obturation was carried out using roll/customized cone technique. Post-endodontic restoration completed with Glass Ionomer Cement, and patient kept under periodic follow-up.
2	11/M	Trauma at 2.5 years; laceration & suturing of the maxillary labial gingiva	8.5 years	Primary tooth trauma with early exfoliation	Delayed eruption + hypoplasia of 11	Delayed eruption with hypoplasia in 11	Initial observation for spontaneous eruption. On failure of eruption, soft tissue surgical exposure of 11 was performed using Diode laser. MBT brackets bonded on adjacent teeth and button attached to 11. Orthodontic extrusion initiated with 2×4 appliance and light eruptive traction. After crown exposure and bracket debonding, pinpoint pulpal exposure managed by Vital Pulp Therapy with Mineral Trioxide Aggregate (MTA), followed by GIC base. Esthetic interim composite restoration done. Clinical and radiographic follow-up performed at 3 months.
3	12/F	Trauma to primary anterior teeth in early childhood with intrusion of teeth	9 years	Intrusion	Enamel Hypoplasia and secondary caries of 11 & 12	Chronic irreversible pulpitis in 11, Dental caries in 12	Clinical and radiographic evaluation of 11 and 12. Root canal treatment initiated for 11 with working length determination, biomechanical preparation, master cone verification, and obturation using bioceramic sealer. Post-endodontic restoration of 11 completed with composite resin. Caries removal and esthetic composite build-up performed for hypoplastic/caries-affected 12. Follow-up clinical and radiographic assessment done at 3 months.
4	9/M	Fall during playing 6 months back	6 months	Palatal luxation of 51 and 52	Delayed eruption of 11	Soft tissue impaction of 11	Clinical and radiographic examination confirmed palatally displaced primary teeth and delayed eruption of 11. Extraction of traumatized primary teeth performed. Periodic monitoring showed eruption bulge of 11. Soft tissue obstruction was managed by operculectomy/surgical exposure using diode laser to uncover the incisal edge and facilitate natural eruption. One-month clinical and radiographic follow-up showed improved eruption.
5	12/F	Intrusion of primary incisors at 3 years of age	9 years	Intrusion + impacted primary tooth	Impaction of 61 and 23 & odontome-like mass	Impacted 61 and 23 with odontome	Clinical and radiographic localization of impacted teeth and radiopaque mass. Full-thickness mucoperiosteal flap reflected, followed by bone guttering and surgical exposure. Removal of retained/intruded primary tooth and odontome-like calcified mass performed. Surgical site irrigated and sutured. Follow-up at 1 week and 3 months demonstrated satisfactory healing and eruption progress.

**Figure 1:**
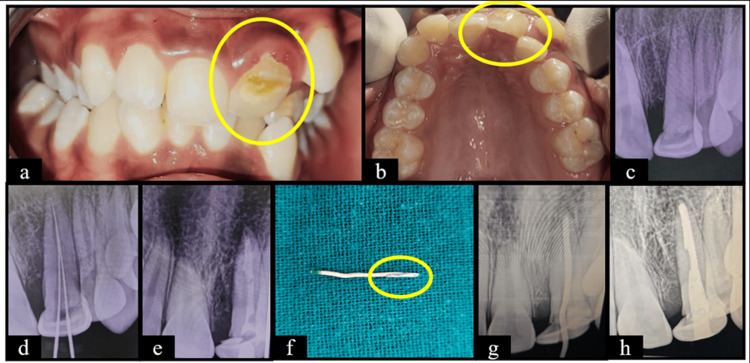
Case one (a) Frontal view showing hypoplastic and dilacerated tooth wrt 21; (b) Maxillary occlusal view; (c) Pre-operative RadioVisioGraphy (RVG) wrt 21; (d) Working length determined wrt 21; (e) Calcium hydroxide dressing given wrt 21; (f) Modified gutta percha by adding 2 pieces at the apical third; (g) Master apical cone wrt 21; (h) Post endodontic restoration done with Glass Ionomer Cement wrt 21. Manufacturer details: Glass Ionomer Cement (3M, St. Paul, MN, USA).

**Figure 2:**
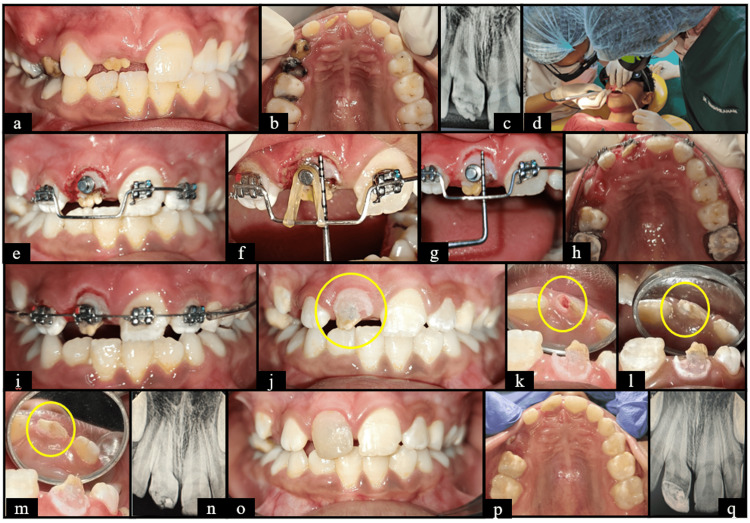
Case two (a) Pre-operative frontal view; (b) Pre-operative maxillary occlusal view; (c) Pre-operative RVG wrt 11; (d) Surgical exposure wrt 11 using Diode laser; (e) Frontal view showing McLaughlin, Bennett, and Trevisi (MBT) brackets wrt 12, 21 and 22 and button wrt 11; (f) Length of the visible portion of crown wrt 11- 6 mm; (2g) Length of the visible portion of crown wrt 11- 8 mm at 48 hours follow up; (h) Maxillary occlusal view showing 2*4 appliance; (i) Frontal view showing 2*4 appliance; (j) Frontal view after bracket debonding and Pulpal exposure seen wrt 11; (k) Pin point pulpal exposure seen after debonding wrt 11; (l) Mineral Trioxide Aggregate (MTA) placed; (m) Glass Ionomer Cement (GIC) placed; (n) RadioVisioGraphy (RVG) wrt 11 after pulp capping; (o) Frontal view at 3 months follow up; (p) Maxillary occlusal view at 3 months follow up; (q) RVG wrt 11 at 3 months follow up. Manufacturer details: Glass Ionomer Cement (3M, St. Paul, MN, USA); Diode Laser (Epic X Diode Laser, BIOLASE, Irvine, CA, USA).

**Figure 3:**
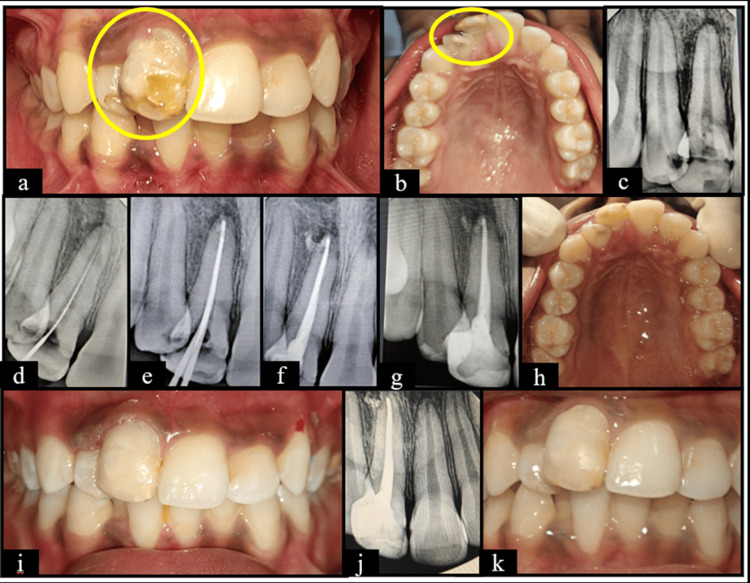
Case three (a) Frontal view showing hypoplastic teeth wrt 11 and 12; (b) Maxillary occlusal view showing hypoplastic teeth wrt 11 and 12; (c) Pre-operative RadioVisioGraphy (RVG) wrt 11 and 12; (d) Working length determined wrt 11; (e) Master Apical Cone RVG wrt 11; (f) Obturation wrt 11 using Bioceramic sealer; (g) Post-operative RVG wrt 11 and 12; (h) Maxillary occlusal view showing composite restoration wrt 11 and 12; (i) Frontal view showing composite restoration wrt 11 and 12; (j) RVG wrt 11 and 12 at 3 months follow up; (k) Frontal view at 3 months follow up. Manufacturer details: Bioceramic Sealer (Meta Adseal Plus, META BIOMED Co., Ltd., Cheongju-si, Korea).

**Figure 4:**
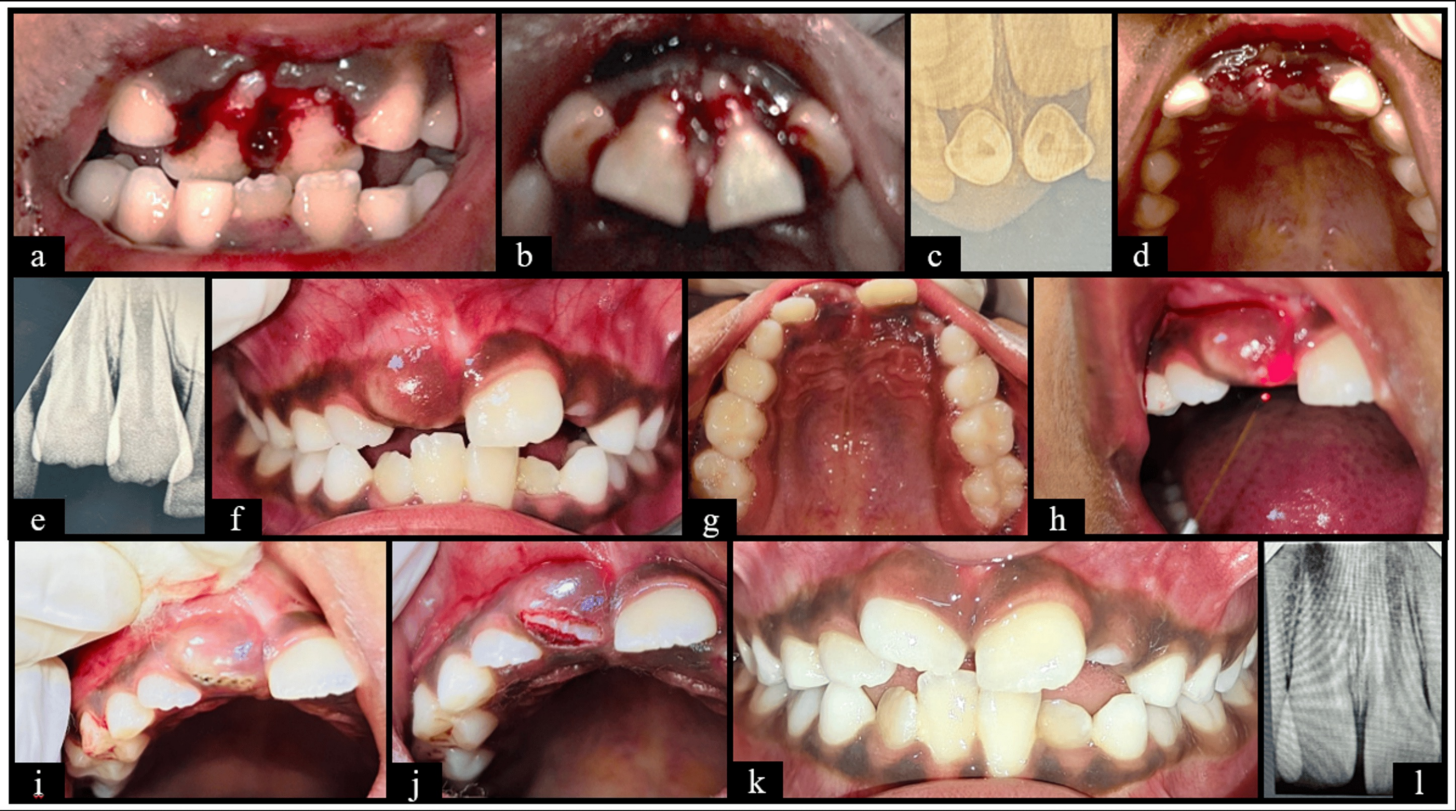
Case four (a) Frontal view showing palatally luxated teeth wrt 51 and 61; (b) Maxillary occlusal view showing palatally luxated teeth wrt 51 and 61; (c) Pre-operative RadioVisioGraphy (RVG) 51 and 61; (d) Maxillary occlusal view after extraction wrt 51 and 61; (e) RVG wrt 11 and 21; (f) Frontal view showing eruption bulge wrt 11 at 6 months follow up; (g) Maxillary occlusal view showing eruption bulge wrt 11 at 6 months follow up; (h-j) Exposure of incisal edge of tooth via Diode laser; (k) Frontal view at 1 month follow up after exposure; (l) RVG wrt 11 and 21 at 1 month follow up after exposure. Manufacturer details: Diode Laser (Epic X Diode Laser, BIOLASE, Irvine, CA, USA).

**Figure 5:**
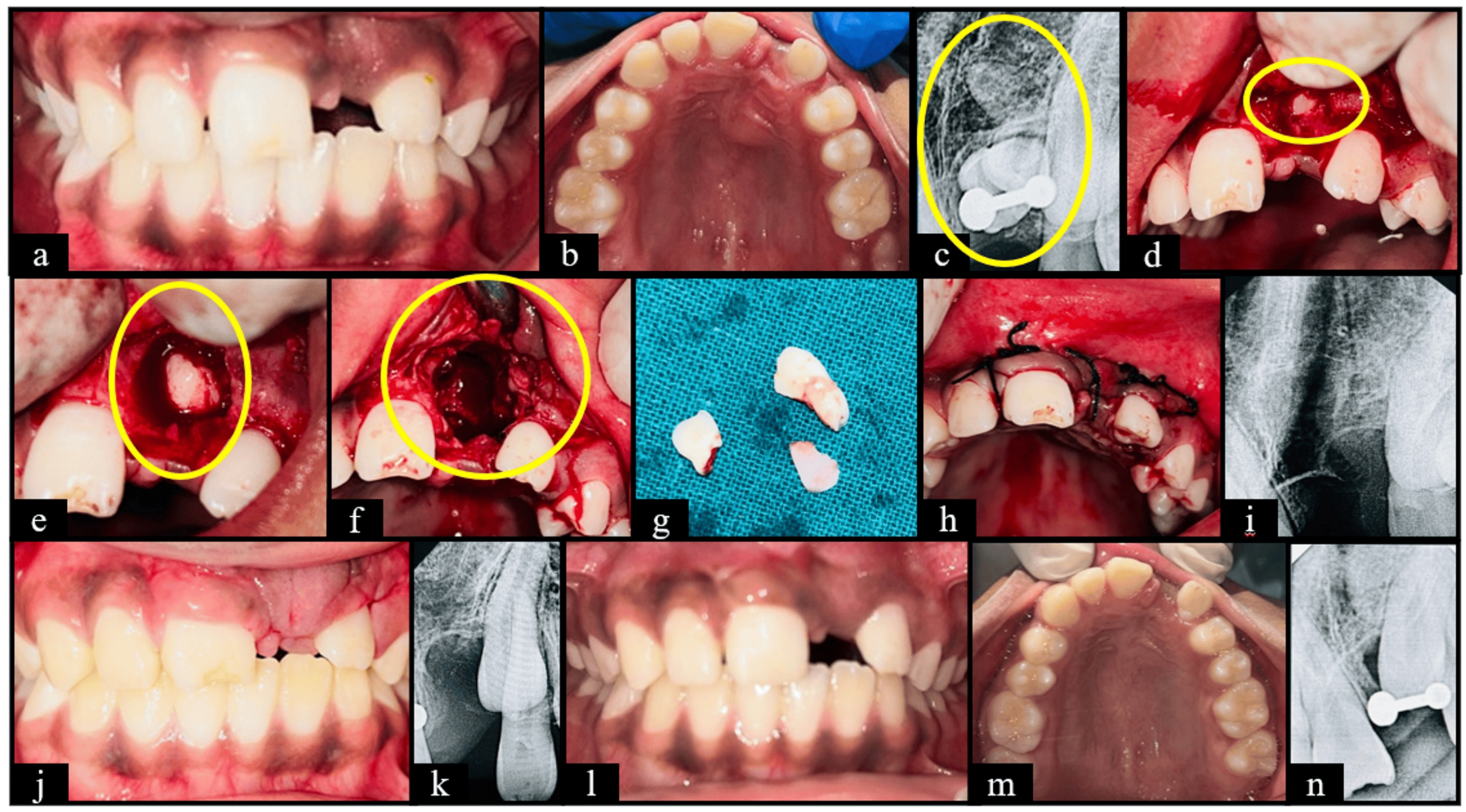
Case five (a) Frontal view showing clinically missing tooth wrt 21; (b) Maxillary occlusal view showing clinically missing tooth wrt 21 and 23; (c) Pre-operative RadioVisioGraphy (RVG) 11, 21 & 22 region showing radiopaque mass wrt 21 region; (d) Flap reflected and bone guttering done; (e) Tooth exposure; (f) Socket after tooth extraction; (g) Extracted supernumerary and intruded primary tooth; (h) After suture placement; (i) RVG wrt 11, 21 & 22 region after extraction; (j) Frontal view at 1 week follow up; (k) RVG wrt 21 at 1 week follow up; (l) Frontal view at 3 months follow up; (m) Maxillary occlusal view at 3 months follow up; (n) RVG wrt 21 at 3 months follow up.

These were patients in the 11 to 13-year age group who reported to the Department of Pediatric and Preventive Dentistry with concerns such as yellowish discoloration, malformed teeth, pain, and delayed eruption. On clinical and radiographic examination, multiple findings were seen, such as enamel hypoplasia (Figures 1-3), crown dilaceration (Figure 1), delayed eruption (Figure 2), impaction, and odontome-like malformations (Figures 4, 5).

All procedures were performed after obtaining informed consent from the parents or guardians, and the diagnostic and treatment protocols adhered to the ethical principles of the Declaration of Helsinki (2008). Clinical histories were obtained from caregivers and available records. Eruption delays were calculated in months from the expected eruption age. Radiographs were independently evaluated by two clinicians.

## Discussion

Traumatic injuries to the primary dentition exert effects on the developing permanent successors due to the intimate anatomical relationship between primary tooth roots and permanent tooth buds. Caeiro-Villasenín et al. (2022) concluded that the principal factors responsible for the nature and severity of the sequelae in the permanent teeth are the type of injury, direction of force, and developmental stage of the permanent tooth bud at the time of trauma [11]. Early sequelae are summarized in Table 2 based on prevalence data reported by Soporowski et al. (1994) [12] and Yılmaz et al. (2021) [13].

**Table 2:** Prevalence of immediate sequelae after trauma to the primary teeth [12,13]

Immediate sequelae (detectable radiographically within 6–12 months)	Prevalence
Inflammatory root resorption	6–14%
Arrested root development	10–15%
Pulp necrosis (primary tooth)	26.3%
Calcific degeneration	10.5%
Ankylosis (primary tooth)	7.9%

Long-term sequelae are presented in Table 3, with prevalence figures derived from regional studies by Tewari et al. (2018) [14] for India, Zilberman et al. (2008) [15] and Bassat et al. (1985) [16] for America, Bardellini et al. (2017) [17] and Costa et al. (2023) [18] for Europe, and Christophersen et al. (2005) for the United Kingdom [19,20]. 

**Table 3:** Prevalence of long-term sequelae in permanent successors after trauma to the primary teeth [14-20]

Long-term sequelae (manifest at eruption, 2–6 years post trauma)	Prevalence in affected successors (%)				Predisposing primary tooth injuries
	India	America	Europe	United Kingdom	
Enamel discoloration/Hypoplasia	16- 52%	15-25%	14.3-25%	9-25%	Intrusion, extrusion, avulsion, complicated fractures
Eruption disturbances	4.7- 10.57%	5-15%	60.7%	5-18%	Intrusion, avulsion (age <4 years)
Root dilaceration	22-41%	1-5%	1.1%	2-8%	Intrusion, severe displacement
Crown dilaceration	17-29%	6-15%	6-26%	6-20%	Intrusive luxation, avulsion, root fractures
Sequestration of entire tooth germ	10%	Rare (<1%)	Not reported	Rare	Intrusion, avulsion
Odontome-like malformation	10-14%	Rare	Not reported	Rare	Intrusive luxation or avulsion

The findings of the present case series are in strong concordance with existing literature. In case one (Figure 1), dilaceration of the crown along with enamel hypoplasia occurred after avulsion of primary maxillary central incisors at the age of three years, similar to the findings by Costa et al. (2023), who reported a prevalence of 25.7% of developmental disturbances in permanent successors, with enamel hypoplasia and dilaceration being the most frequent outcomes after avulsion of primary teeth [18]. Direct mechanical displacement leading to deformation of the developing tooth germ is the main cause of these defects. A high prevalence of crown anomalies following avulsion of primary incisors was also reported by Yilmaz et al. (2021) and it necessitates definitive endodontic and restorative management, as was done in the present case [13].

Delayed eruption and enamel hypoplasia secondary to early exfoliation and palatal luxation respectively as seen in cases two and four (Figures 2, 4) are also in concordance with the findings reported by Alfarraj et al. (2022) [21] and (Project for Bachelor’s degree: Hussein RA. Delayed Tooth Eruption; 2023). They demonstrated the effectiveness of diode laser-assisted surgical exposure in managing eruption disturbances caused by soft tissue barriers followed by orthodontic and restorative modalities.

Intrusion of the primary tooth, which resulted in enamel hypoplasia and increased susceptibility to caries in permanent successor was observed in case three (Figure 3). Altun et al. (2009) [22] and Bardellini et al. (2017) [17] reported that developmental enamel defects occur following intrusion of primary teeth. Further, Disha et al. (2024) also showed the increased risk of secondary caries which further requires intervention [23].

In case five (Figure 5), intrusion of primary incisors resulted in impaction of the permanent successor along with an odontome-like malformation. Such odontogenic anomalies occur due to compressive injury to the developing tooth germ and dental lamina (Shaked et al. (2008) [24]). Manjula et al. (2014) recommends surgical removal as the treatment of choice to facilitate eruption and prevent further complications [25].

Overall, avulsion predominantly results in crown dilaceration and enamel hypoplasia, while intrusive luxation causes enamel defects, eruption disturbances, and odontogenic malformations [26]. The management approaches commonly used are endodontic therapy, restorative therapy, laser-assisted surgical exposure followed by orthodontic extrusion, and removal of odontogenic malformations. These aim to facilitate eruption, preserve pulpal health, and prevent any secondary complications [27].

This case series has inherent limitations due to its descriptive design, small sample size, and single-center setting, which may limit generalizability. Trauma histories were obtained retrospectively from caregivers and records, introducing possible recall bias. Selection bias may also be present, as only patients with clinically evident sequelae who reported for treatment were included. In this series, enamel hypoplasia was seen in four cases, eruption disturbance/impaction in three cases, and two cases required endodontic treatment.

According to the American Academy of Pediatric Dentistry (AAPD) and International Association of Dental Traumatology (IADT), long-term clinical and radiographic follow-ups after primary tooth trauma are essential because developmental sequelae in permanent successors may present months or years after the initial injury and therefore, are detected only through periodic monitoring [28].

## Conclusions

Traumatic injuries to the primary dentition may result in delayed developmental sequelae in permanent successors, many of which remain undetected until eruption. These disturbances may involve structural defects, eruption abnormalities, or pulpal complications requiring timely diagnosis and care. This case series highlights the diverse presentations of such post-traumatic outcomes.

The present report emphasizes the importance of long-term clinical and radiographic follow-up for early detection and prompt intervention. Timely management can improve functional, esthetic, and psychological outcomes in affected children. It also highlights the need for injury-specific treatment guided by evidence-based protocols.

## References

[1] Ritwik P, Massey C, Hagan J (2015). Epidemiology and outcomes of dental trauma cases from an urban pediatric emergency department. Dent Traumatol.

[2] Petersson EE, Andersson L, Sörensen S (1997). Traumatic oral vs non-oral injuries. Swed Dent J.

[3] Andersson L (2013). Epidemiology of traumatic dental injuries. J Endod.

[4] Petti S, Glendor U, Andersson L (2018). World traumatic dental injury prevalence and incidence, a meta-analysis-one billion living people have had traumatic dental injuries. Dent Traumatol.

[5] Patnana AK, Chugh A, Chugh VK, Kumar P, Vanga NR, Singh S (2021). The prevalence of traumatic dental injuries in primary teeth: a systematic review and meta-analysis. Dent Traumatol.

[6] Goswami M, Aggarwal T (2021). Prevalence of traumatic dental injuries among 1- to 14-year-old children: a retrospective study. Int J Clin Pediatr Dent.

[7] Tewari N, Mathur VP, Siddiqui I, Morankar R, Verma AR, Pandey RM (2020). Prevalence of traumatic dental injuries in India: a systematic review and meta-analysis. Indian J Dent Res.

[8] Fried I, Erickson P, Schwartz S, Keenan K (1996). Subluxation injuries of maxillary primary anterior teeth: epidemiology and prognosis of 207 traumatized teeth. Pediatr Dent.

[9] da Costa VP, Almeida FV, Demarco GT, da Motta MG, Langlois CO, Silva AE, Goettems ML (2026). Impact of traumatic injuries to primary teeth on the development of permanent dentition: findings from a retrospective cohort. Dent Traumatol.

[10] Lopes TS, Santin GC, Marengoni LA, Crispim JB, Ceron LC, Fracasso MLC (2019). Clinical and radiographic sequelae in primary teeth due to dental trauma. Pesqui Bras Odontopediatria Clin Integr.

[11] Caeiro-Villasenín L, Serna-Muñoz C, Pérez-Silva A, Vicente-Hernández A, Poza-Pascual A, Ortiz-Ruiz AJ (2022). Developmental dental defects in permanent teeth resulting from trauma in primary dentition: a systematic review. Int J Environ Res Public Health.

[12] Soporowski NJ, Allred EN, Needleman HL (1994). Luxation injuries of primary anterior teeth--prognosis and related correlates. Pediatr Dent.

[13] Yilmaz N, Erbek SM, Reis T, Güdük OF, Baygin O, Tüzüner T (2021). Traumatic dental injuries occurred in primary teeth and their sequel effects on the developmental permanent successors: a controlled study. Pesqui Bras Em Odontopediatria E Clínica Integrada.

[14] Tewari N, Mathur VP, Singh N, Singh S, Pandey RK (2018). Long-term effects of traumatic dental injuries of primary dentition on permanent successors: a retrospective study of 596 teeth. Dent Traumatol.

[15] Zilberman Y, Fuks A, Ben Bassat Y, Brin I, Lustmann J (1986). Effect of trauma to primary incisors on root development of their permanent successors. Pediatr Dent.

[16] Bassat BY, Brin I, Fuks A, Zilberman Y (1985). Effect of trauma to the primary incisors on permanent successors in different developmental stages. Pediatr Dent.

[17] Bardellini E, Amadori F, Pasini S, Majorana A (2017). Dental anomalies in permanent teeth after trauma in primary dentition. J Clin Pediatr Dent.

[18] Costa MP, Jural LA, Silva LL (2023). A 14-year follow-up study of sequelae in primary teeth and permanent successors after dental trauma. Pesqui Bras Odontopediatria Clín Integr.

[19] do Espírito Santo Jácomo DR, Campos V (2009). Prevalence of sequelae in the permanent anterior teeth after trauma in their predecessors: a longitudinal study of 8 years. Dent Traumatol.

[20] Christophersen P, Freund M, Harild L (2005). Avulsion of primary teeth and sequelae on the permanent successors. Dent Traumatol.

[21] Alfarraj JH, Alsaif F, Alsaad SA (2022). Management of delayed eruption in permanent incisor following intrusion injury of primary dentition: a case report. Int Med Case Rep J.

[22] Altun C, Cehreli ZC, Güven G (2009). Traumatic intrusion of primary teeth and its effects on the permanent successors: a clinical follow-up study. Oral Surg Oral Med Oral Pathol Oral Radiol Endod.

[23] Disha V, Zaimi M, Petrela E, Aliaj F (2024). An investigation into the prevalence of enamel hypoplasia in an urban area based on the types and affected teeth. Children (Basel).

[24] Shaked I, Peretz B, Ashkenazi M (2008). Development of odontoma-like malformation in the permanent dentition caused by intrusion of primary incisor--a case report. Dent Traumatol.

[25] Manuja N, Nagpal R, Singh M, Chaudhary S (2011). Management of delayed eruption of permanent maxillary incisor associated with the presence of supernumerary teeth: a case report. Int J Clin Pediatr Dent.

[26] Costa V, da Silva-Júnior I, Shqair A (2018). Fusion of permanent teeth as post-traumatic sequelae of trauma in primary dentition: a case report with fifteen years of follow-up. J Clin Exp Dent.

[27] Kermanshah H, Najafrad E, Valizadeh S (2022). Forced eruption: alternative treatment approach to restore teeth with subgingival structure. Case Rep Dent.

[28] Day PF, Flores MT, O'Connell AC (2020). International Association of Dental Traumatology guidelines for the management of traumatic dental injuries: 3. Injuries in the primary dentition. Dent Traumatol.

